# Computational design and geometry-driven modeling of TiN-based plasmonic metasurface absorbers

**DOI:** 10.1038/s41598-026-43764-6

**Published:** 2026-04-02

**Authors:** Ahmed Nagaty, Arafa H. Aly, Walied Sabra

**Affiliations:** TH-PPM Group, Physics Department, Faculty of Science, Beni-Suef University, Beni Suef, Egypt

**Keywords:** Metasurface, TiN, Plasmonics, Broadband absorption, Nanoantennas, Tunability, Thermal applications, Engineering, Materials science, Nanoscience and technology, Optics and photonics, Physics

## Abstract

We numerically investigate plasmonic metasurface absorbers based on hollow square, hollow cylindrical, and conical nanoantenna geometries fabricated from various plasmonic materials, with emphasis on titanium nitride (TiN) as a refractory alternative to noble metals. Full-wave finite-element simulations in the visible range reveal geometry- and material-dependent absorption behavior, where TiN designs exhibit strong broadband absorption and reduced sensitivity to geometrical variations within the studied parameter space. Fitted analytical models are developed to describe the dependence of wavelength-averaged absorption (400–800 nm) on key structural parameters, enabling reliable performance prediction and efficient optimization. The results show that the intrinsic optical losses and damping properties of TiN support broadband absorption and fabrication tolerance for the examined geometries. This work provides a quantitatively supported framework for designing optimized plasmonic metasurface absorbers for applications such as solar energy harvesting, optical sensing, photothermal conversion, and radiative thermal management.

## Introduction

The controlled manipulation of electromagnetic wave propagation has become a central objective in modern photonics. Precise engineering of phase, amplitude, and polarization responses enables tailored light–matter interaction through mechanisms such as impedance matching, resonant mode hybridization, and cavity-assisted confinement. A key advancement in this context is the development of metamaterials—artificially structured media composed of subwavelength unit elements whose collective response can be designed to achieve effective optical properties unattainable in naturally occurring materials^1,2^. Owing to their distinctive electromagnetic properties, metamaterials have facilitated a wide range of applications, including negative refractive index media^3^, electromagnetic cloaking devices^4^, structural color filtering systems^5^, and sub-diffraction-limit imaging technologies^6^. Metasurfaces, a two-dimensional subclass of metamaterials, have attracted considerable interest because they enable precise control of electromagnetic waves using subwavelength building blocks within an ultrathin platform^7,8^. In contrast to bulk metamaterials, metasurfaces consist of planar arrays of engineered resonant nanoelements whose geometry and spatial distribution determine their optical response. By tailoring these structural parameters, metasurfaces can be designed to achieve controlled wavefront manipulation^9^, polarization management^10^, and efficient holographic functionality^11^, while maintaining reduced thickness and fabrication compatibility.

Metasurfaces have emerged as powerful platforms for engineering highly efficient electromagnetic absorbers. In this context, a perfect absorber refers to a structured configuration capable of nearly complete dissipation of incident radiation, while suppressing both reflection and transmission channels^12^. Several design strategies have been explored to approach unity absorption, including nanostructured metallic interfaces^13^, subwavelength perforated architectures^14^, and multilayer resonant cavities^15^. More recently, plasmonic metasurfaces exploiting localized surface plasmon resonances (LSPRs) have demonstrated enhanced spectral tunability and broadband absorption performance, owing to their strong field confinement and controllable dissipative losses^16,17^.

In addition to optical absorption applications, plasmonic metasurfaces have been explored for their potential in sensing and imaging technologies. By exploiting the strong field enhancement at plasmonic resonances, these structures enable highly sensitive detection mechanisms suitable for biosensing, chemical analysis, and environmental monitoring^18,19^. The ability to engineer metasurfaces designs with tunable spectral responses allows for multi-functional devices that integrate absorption and sensing capabilities within a compact platform. Moreover, metasurfaces have shown promise in photothermal therapy, where controlled light absorption can be used for localized heating in biomedical applications^20,21^.

Plasmonic metasurface absorbers also play a significant role in energy conversion and thermal management technologies. Their ability to tailor absorption within selected spectral bands makes them particularly attractive for applications including thermophotovoltaic systems, infrared camouflage, and passive radiative cooling^22,23^. By carefully adjusting geometrical parameters—such as antenna height, cavity dimensions, and tapering profiles—the spectral position, bandwidth, and magnitude of absorption can be systematically controlled, enabling improved energy-harvesting performance in solar and thermophotonic platforms^24,25^. Importantly, the absorption response is governed by the interplay between intrinsic material losses and geometry-dependent resonant mode formation, including impedance matching and cavity-assisted plasmonic confinement. Achieving optimized performance therefore requires a balanced design strategy that integrates material dispersion characteristics with structural configuration to ensure broadband efficiency and spectral stability within the targeted wavelength range.

Among the different plasmonic materials, titanium nitride (TiN) has emerged as a compelling alternative to traditional noble metals such as gold (Au) and silver (Ag) due to its superior thermal stability, CMOS compatibility, and strong plasmonic response in the visible to near-infrared spectrum^26,27^. TiN-based metasurfaces have demonstrated remarkable absorption properties, making them suitable for applications in photodetectors, energy harvesting, and thermal management systems^28,29^.

In this study, we propose a systematic approach for designing plasmonic metasurfaces absorbers with tunable absorption properties using TiN and other plasmonic nanostructures such as Au, Ag and Al. Here, we present a wide comparison between using these metallic nanoantennas to the alternative plasmonic TiN nanoantennas. By employing numerical simulations and analytical modeling, we explore the role of nanoantenna geometry, material composition, and structural parameters in optimizing absorption efficiency. The findings of this work contribute to the development of high-performance absorbers for applications in sensing, photonics, and optoelectronic devices. In contrast to our previous investigations of solid nanodisk and rectangular metasurface absorbers, the present study examines hollow square, hollow cylindrical, and conical nanoantenna geometries, which introduce additional structural degrees of freedom through internal voids and tapered profiles. These configurations enable more flexible control of plasmonic mode distribution, field confinement, and resonance hybridization. Moreover, beyond conventional parametric sweeps, this work establishes geometry–absorption correlation maps across multiple plasmonic materials and develops fitted predictive models that allow direct estimation of average absorptance from structural parameters within the investigated range. This integrated geometry-oriented and model-supported framework extends the design methodology beyond purely empirical optimization approaches.

Recently, inverse-design methodologies have emerged as powerful tools for metasurface absorber optimization, enabling automated retrieval of geometric configurations that meet predefined spectral objectives. Unlike conventional forward parametric sweeps, inverse approaches—based on adjoint optimization, machine learning, or neural-network-assisted regression—directly map desired absorption characteristics to structural parameters. Such strategies have demonstrated accelerated convergence and enhanced design flexibility for broadband and multifunctional absorbers^30,31^. In this context, the present work complements inverse-design paradigms by providing geometry–absorption correlation maps and predictive polynomial models that enable rapid performance estimation within physically constrained design intervals. This semi-analytical, geometry-oriented framework offers a computationally efficient alternative while remaining compatible with future integration into data-driven inverse optimization workflows.

## Theoretical treatment

**Fig. 1:**
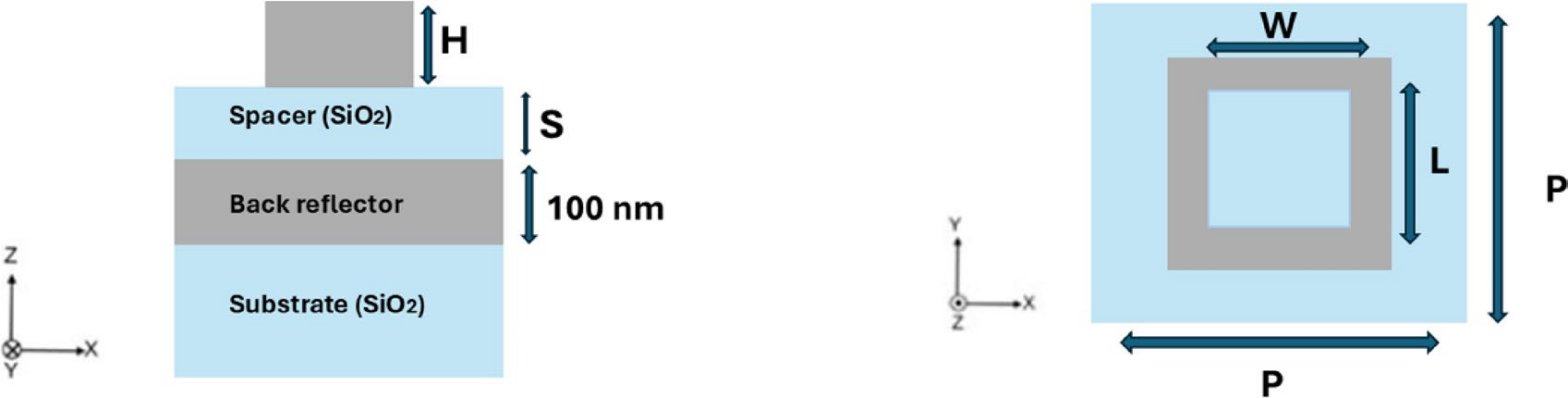
Schematic diagrams of side and top views of the unit cell of the proposed MSF structures.

Figure 1 illustrates the top and cross-sectional views of the proposed metasurface (MSF) unit cell. The structure comprises a bi-periodic array of hollow nanoantennas placed above a SiO₂ dielectric spacer and backed by a 100 nm thick metallic reflector to eliminate transmission and ensure reflection-mode operation. The nanoantennas are fabricated from Al, Au, Ag, and TiN to enable material-dependent performance comparison. The SiO₂ refractive index is adopted from Ref.^32^, while the dispersive permittivity of Al, Au, and Ag follows the Brendel–Bormann model reported in Ref.^33^. The dielectric function of TiN is based on experimentally measured data as described in Ref.^34^. To investigate geometry-driven absorption behavior, three nanoantenna configurations are considered: hollow square, hollow cylindrical, and conical designs. These geometries provide additional control over plasmonic mode confinement and impedance matching within the visible spectral range. The metasurface is illuminated under normal incidence with a plane wave spanning 400–800 nm. Broadband absorption originates from the combined effects of localized plasmonic resonances in the nanoantennas, cavity-assisted interference within the spacer layer, and intrinsic material losses.

The optical properties of the proposed metasurface designs are investigated using a three-dimensional finite-element method (FEM) solver (COMSOL Multiphysics, Wave Optics Module). The simulations employ a maximum mesh size of 84 nm, with periodic boundary conditions applied along the x- and y-directions. The unit cell period (P), spacer thickness (S), and nanoantenna height (H) are set to 300 nm, 60 nm, and 30 nm, respectively. These values were determined through a parametric sweep to identify the optimal configuration for achieving enhanced optical performance. In this study, we extend our investigation of plasmonic metasurface absorbers to include a hollow square antenna design. A systematic mesh convergence study was conducted to verify the numerical accuracy and stability of the simulated absorptance spectra. The mesh density was progressively refined, and the maximum element size was reduced until the variation in calculated absorption remained below 1%, ensuring reliable and mesh-independent results. Furthermore, the selected geometric parameter ranges were carefully determined to maintain compatibility with realistic nanofabrication constraints while still enabling efficient tuning of plasmonic resonances within the visible wavelength region.

## Results and discussion

### Hollow square metasurface

In this study, the principal figure-of-merit is defined as the wavelength-averaged absorptance across the visible spectral range (400–800 nm). This averaged quantity is employed to systematically assess and compare the broadband absorption efficiency of the investigated metasurface configurations.

**Fig. 2:**
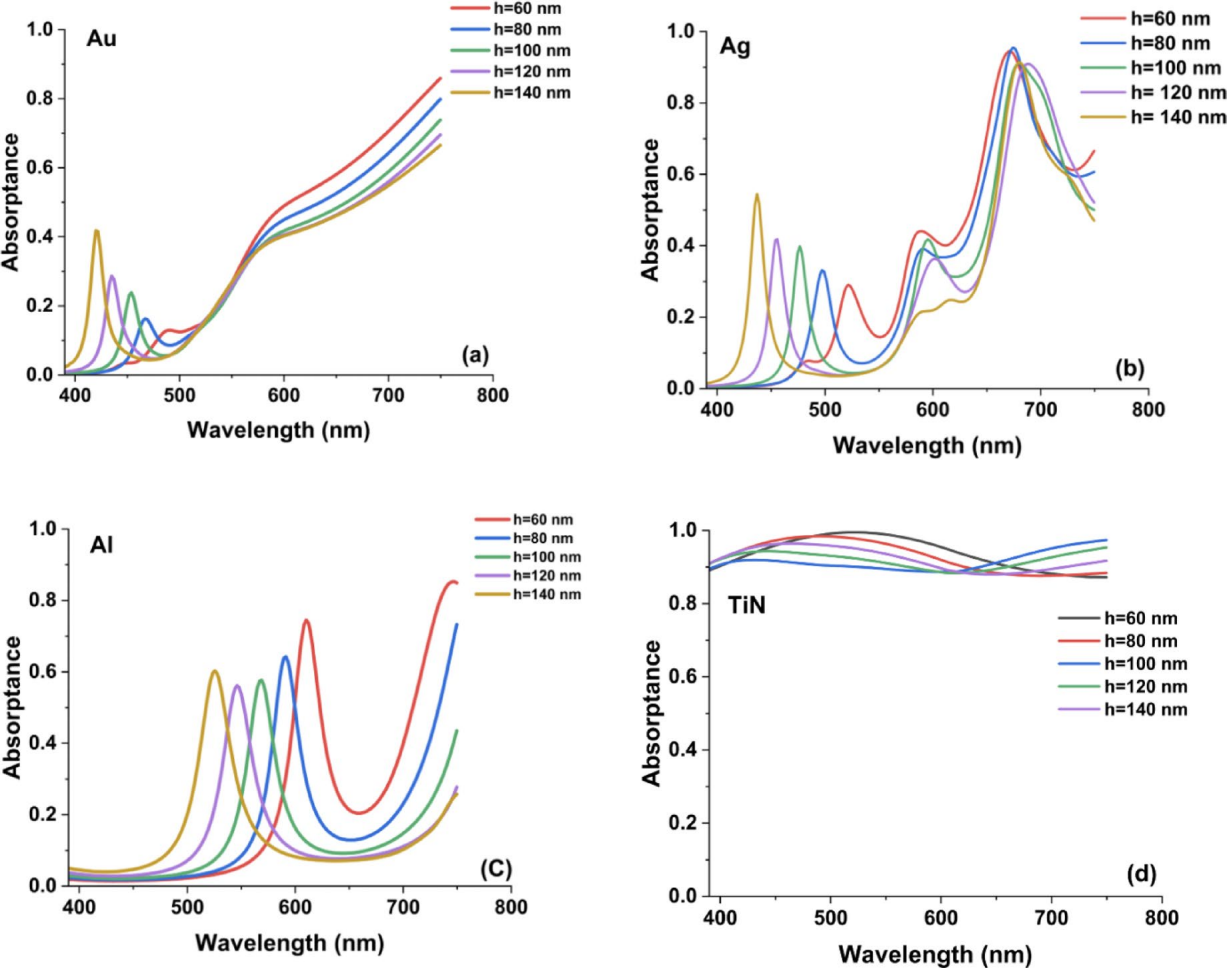
The resulted absorption spectra in the case of hollow square metasurface designs by changing the height by 20 nm step from H = 60 to 140 nm.

Figure 2 presents the calculated absorptance spectra of metasurfaces incorporating hollow square nanoantennas for varying antenna heights. Introducing a hollow square architecture provides additional geometric degrees of freedom relative to conventional solid nanodisks or rectangular antennas. Specifically, the presence of an internal void modifies the effective modal distribution and enables enhanced control over vertical field confinement and resonance hybridization. These structural features influence plasmonic mode formation, field redistribution within the cavity-like region, and the overall coupling behavior, thereby affecting both resonance position and broadband absorption performance.

From a physical standpoint, increasing the antenna height enhances the impedance matching between free space and the metasurface, thereby reducing reflection losses. At the same time, a taller structure promotes stronger coupling between localized surface plasmon resonances and cavity-like modes supported within the hollow region. The presence of the internal void alters the effective dielectric environment of the resonator, which intensifies electromagnetic field confinement and contributes to an expanded absorption bandwidth. The curves indicate that the antenna height has a pronounced impact on the absorption characteristics. For the Au- and Ag-based metasurfaces, a pronounced and spectrally confined absorption peak appears within the visible range, located approximately at 500–600 nm for Au and 600–750 nm for Ag, particularly at reduced antenna heights. In contrast, TiN-based metasurfaces do not exhibit a distinct narrow-band resonance over this spectral window; instead, they demonstrate a comparatively broader and smoother absorption profile across the same wavelength range. As the height increases, the following aspects are observed:

- The red-shift observed with increasing nanoantenna height is not primarily governed by enhanced lateral near-field coupling between adjacent elements. In fact, increasing the height tends to reduce lateral coupling due to the larger effective separation in the dominant interaction plane. Instead, the resonance behavior is mainly dictated by the formation of vertically confined, longitudinal cavity-like plasmonic modes along the nanoantenna axis. As the antenna height increases, the effective modal confinement length along the vertical direction is extended, which lowers the resonance energy and consequently shifts the resonance toward longer wavelengths. This mechanism is consistent with Fabry–Pérot-like plasmonic resonances reported in vertically extended nanostructures, where the resonance condition is directly linked to the effective optical length of the confined mode. Thus, the height-dependent red-shift originates from vertical field redistribution and longitudinal mode formation rather than from strengthened lateral plasmonic coupling. As the antenna height increases, the effective longitudinal modal length becomes larger, which lowers the resonance energy and produces a corresponding red-shift in the resonant wavelength. Although increasing height reduces lateral near-field coupling between adjacent elements—an effect that could favor a blue-shift—the optical response in this case is predominantly governed by vertically confined, cavity-like plasmonic modes. The elongation of these longitudinal modes with increasing height outweighs the reduced lateral coupling, resulting in an overall red-shift of the resonance. Furthermore, the evolution of absorption amplitude with antenna height does not follow a universal trend; instead, it strongly depends on the intrinsic optical properties and damping characteristics of the specific material. For lossy plasmonic materials such as TiN, increasing the antenna height enhances the effective interaction volume and supports stronger confinement of resonant fields within the hollow structure. Because TiN exhibits a large imaginary permittivity in the visible range, this enhanced confinement leads to increased dissipative absorption. In contrast, aluminum does not follow this trend due to its optical response in the visible range, which is characterized by a large negative real permittivity and comparatively lower loss. As a result, increasing antenna height in Al-based metasurfaces does not necessarily increase absorption and may instead enhance radiative scattering or shift the resonance away from the strongest-loss region. The non-monotonic absorption behavior observed for Al therefore does not contradict the simulation results but rather highlights the importance of material-specific plasmonic damping and dispersion in determining absorption amplitude trends
- *Amplitude variation*: The absorption amplitude tends to increase with increasing the antenna height, suggesting that a larger volume of the plasmonic material enhances the electromagnetic field confinement within the hollow region. The enhanced confinement leads to stronger plasmonic excitation and, consequently, higher average absorption. This observation is in line with previous studies on TiN-based plasmonic metasurfaces, which have shown improved performance with optimized structural dimensions.
- *Bandwidth broadening*: In addition to the resonance shift, the absorption bandwidth appears to broaden slightly with increasing antenna height. The broader spectral response may be linked to a combination of increased radiative damping and modified near-field interactions in the hollow square geometry. The effect is similar to that seen in metasurfaces where the coupling between discrete plasmonic elements induces a more complex spectral profile. The near-unity absorption can be understood as a consequence of efficient impedance matching between the metasurface and free space at the resonant condition, which suppresses reflection losses. This effect originates from strong resonant coupling between the TiN nanoantenna and the dielectric spacer layer, resulting in pronounced electromagnetic field confinement and enhanced energy dissipation within the intrinsically lossy TiN medium.
- Compared with our previously reported nanodisk and rectangular metasurface configurations^35^, the hollow square nanoantenna architecture demonstrates noticeably different spectral characteristics. In earlier geometries, systematic variations in disk diameter or rectangle side length resulted in predictable shifts of well-defined resonance peaks. In contrast, the hollow square configuration introduces an extra structural degree of freedom through its central cavity, which modifies the local dielectric environment and alters the current distribution within the antenna. This internal void facilitates the excitation of additional plasmonic modes and resonance hybridization, thereby expanding the achievable tuning range. Such multimodal behavior enhances design flexibility and is particularly beneficial for applications requiring spectral selectivity across multiple bands or extended broadband absorption, including multi-band sensing and solar energy harvesting. The observed improvements in absorption with increasing antenna height, combined with the inherent flexibility of the hollow square design, suggest that such metasurfaces can be tailored to achieve near-unity absorption over a broader spectral range. This tunability is particularly important for applications in thermal management and plasmonic sensing, where control over the spectral response is critical. Moreover, the hollow design may offer advantages in terms of reduced fabrication complexity or enhanced thermal stability, as the reduced metal filling fraction can alleviate issues related to heat accumulation.

**Fig. 3:**
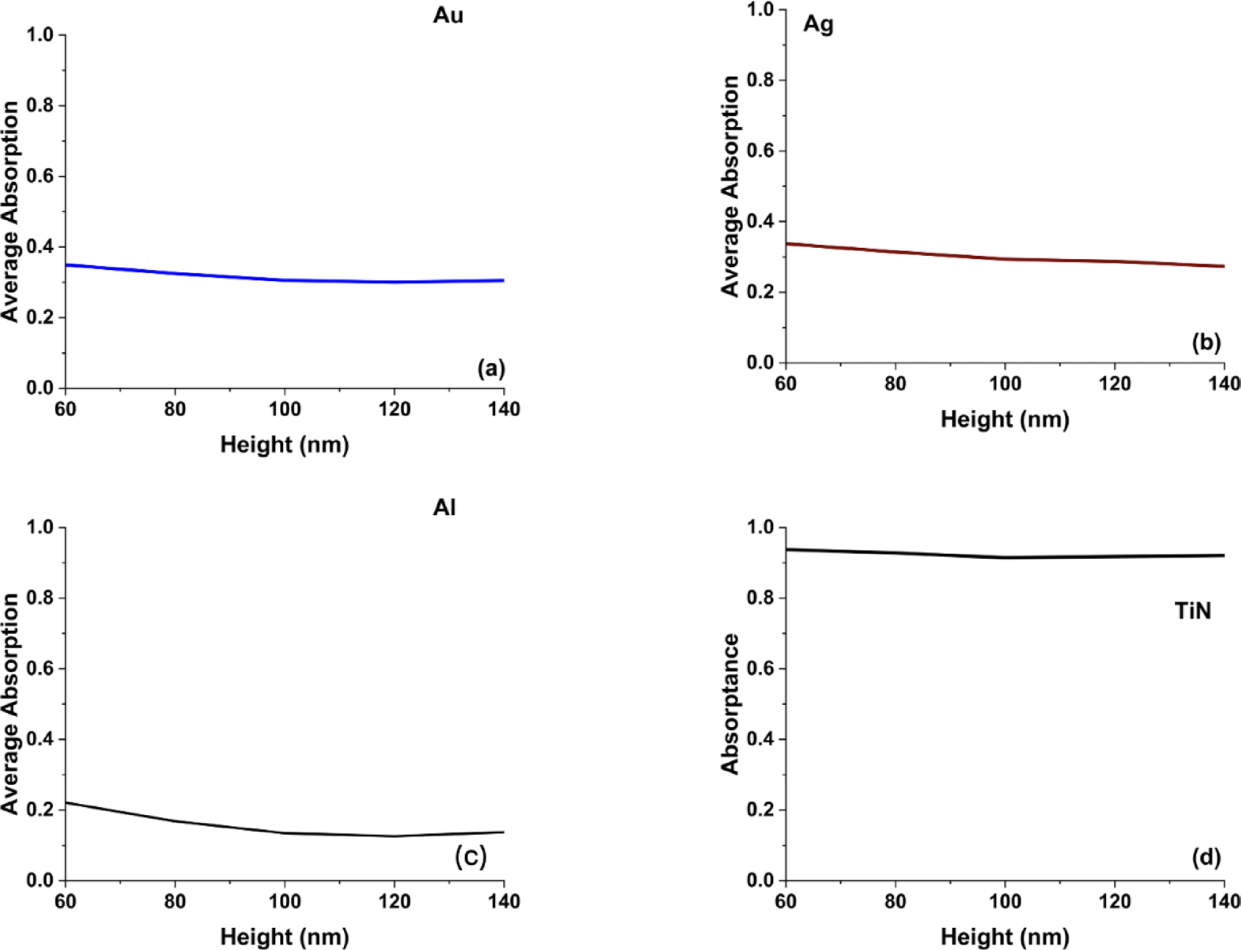
The calculated average absorption curves for hollow square metasurface designs in the case of using (**a**) Au, (**b**) Ag, (**c**) Al, and (**d**) TiN nanoantennas.

As shown in Fig. 3, The average absorption curves for the different plasmonic materials (Au, Ag, Al, and TiN) as a function of antenna height provide key insights into the effect of geometrical variations on plasmonic behavior. TiN maintained stable absorption over varying heights, reflecting its dimensional tolerance and reliable performance across broadband spectra. TiN’s minimal dependence on height can be attributed to its strong plasmonic resonance in the visible and near-infrared spectrum, coupled with its high-loss characteristics that enhance optical absorption. In contrast, Au and Ag exhibit a slight but noticeable decrease in average absorption as height increases, indicating a redshift in plasmonic resonance. The reduction in absorption for Ag is more gradual compared to Au, which suggests that silver’s surface plasmon resonance is more stable against height variations, making it suitable for applications requiring narrowband optical control.

In contrast to TiN, aluminum exhibits a noticeable reduction in absorption as the antenna height increases, reflecting its comparatively weaker plasmonic response within the visible spectrum. This behavior indicates that the absorption trend with height is not universal, but strongly dependent on the intrinsic optical properties of the metal. For Al, the combination of a high plasma frequency and relatively low intrinsic loss in the visible range limits efficient dissipative absorption, and increasing the antenna height tends to enhance radiative scattering rather than absorption. As a result, its performance becomes more sensitive to geometric variations, which constrains its suitability for broadband visible absorbers, although it remains attractive for low-cost UV plasmonic and color-filtering applications.

The overall trend is consistent with prior studies on metasurface absorbers, where increasing structural height modifies resonant mode distributions and can reduce absorption efficiency in noble metals such as Au, Ag, and Al due to altered impedance matching and increased radiative loss. By comparison, TiN maintains broadband absorption with reduced sensitivity to height variation. This robustness originates from its higher intrinsic loss and stronger dissipative damping in the visible regime, which support broadband plasmonic excitation and mitigate sharp resonance shifts. Consequently, TiN-based metasurfaces demonstrate improved tolerance to geometric deviations, making them particularly promising for applications requiring thermal stability and structural reliability, including high-temperature photonics, thermal emitters, and energy-harvesting systems.

Furthermore, the variation in absorption amplitude with antenna height is clearly material dependent. While TiN benefits from enhanced field confinement and increased dissipative loss as the structure becomes taller, aluminum does not follow the same mechanism due to its distinct permittivity characteristics in the visible range. This fundamental difference explains the deviation observed for Al-based metasurfaces and supports the revised, material-specific interpretation provided in the manuscript.

### Hollow cylinder metasurface

**Fig. 4:**
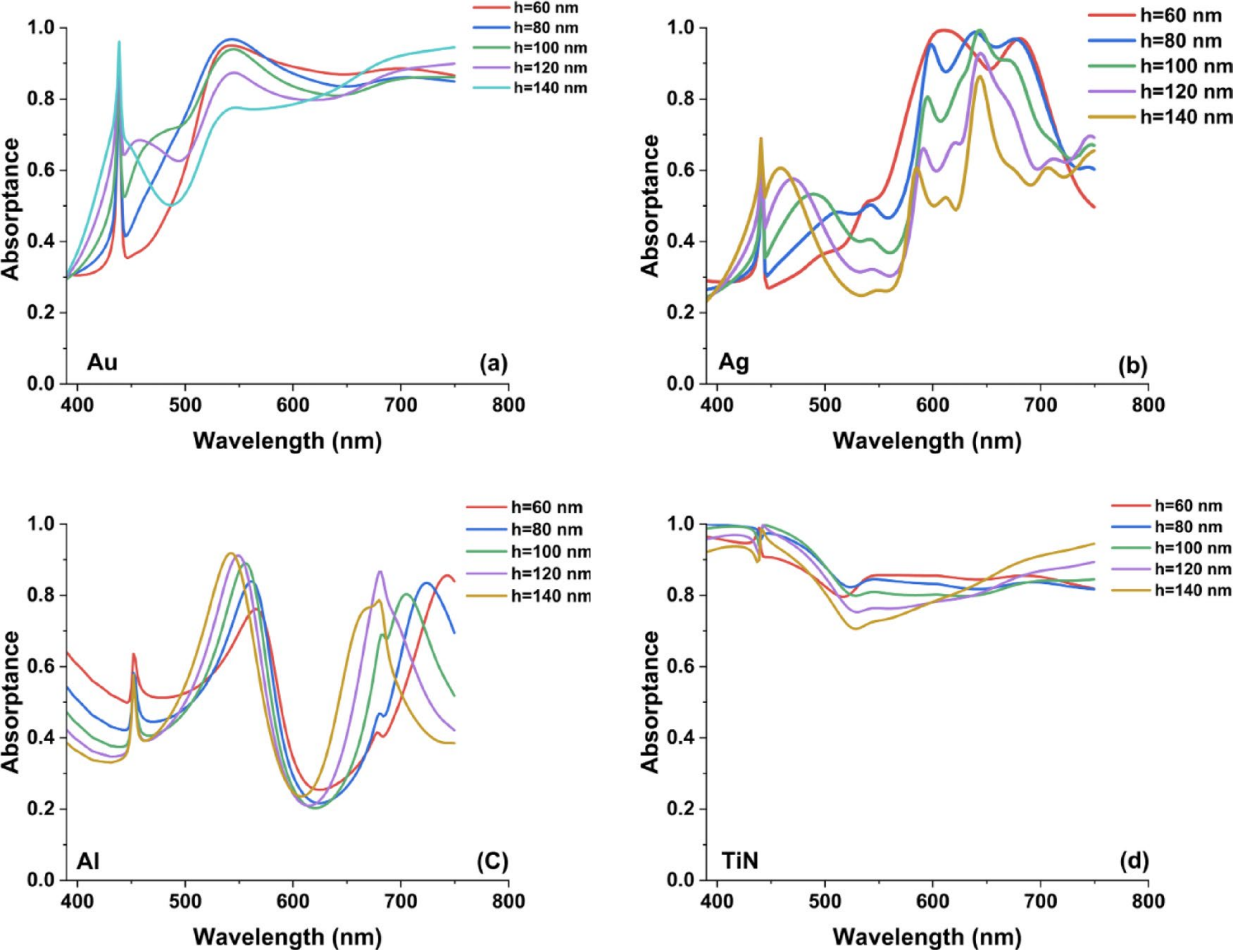
The resulted absorptance spectra in the case of hollow cylinder metasurface designs by changing the height by 20 nm step from H = 60 to 140 nm.

The absorption spectra for the hollow cylinder antenna metasurface using Au, Ag, Al, and TiN reveal key insights into the influence of antenna height on plasmonic resonance and absorption efficiency as presented in Fig. 4.

Among these materials, Titanium Nitride (TiN) demonstrates the highest and most stable absorption across all heights, maintaining values close to unity. This broadband absorption with minimal height dependency can be attributed to TiN’s strong plasmonic resonance and high-loss characteristics, making it a promising candidate for thermal emitters, energy harvesting, and high-temperature applications . In contrast, Au and Ag exhibit pronounced resonance peaks that shift with increasing height, indicating strong localized surface plasmon resonance (LSPR) effects. Gold shows its strongest absorption within the 450–600 nm range, while silver peaks around 600–750 nm, suggesting their potential for wavelength-selective optical applications. The broadening of peaks in these materials at greater heights suggests increased coupling between localized and propagating plasmon modes, which can be useful for tunable photonic devices.

**Fig. 5:**
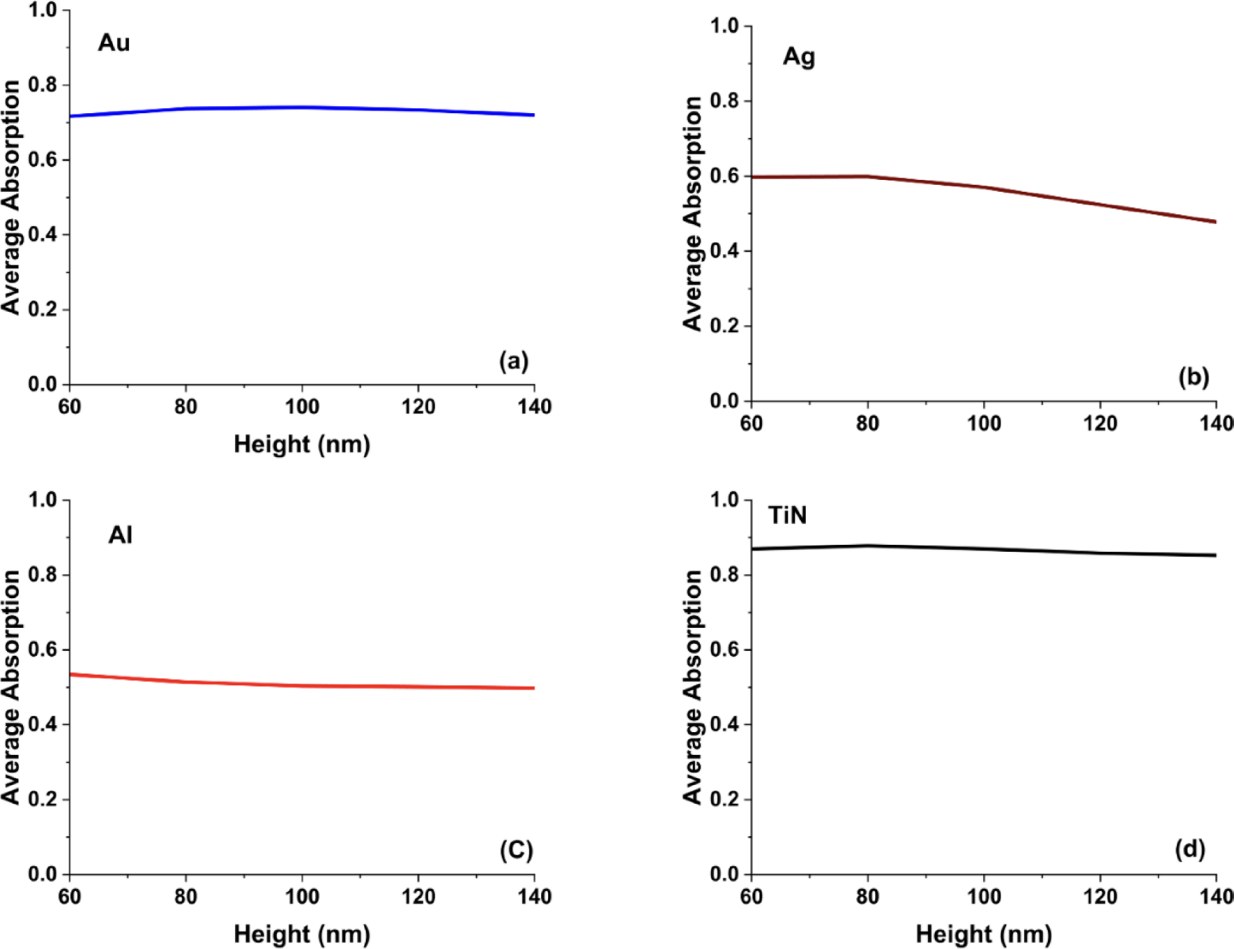
The calculated average absorption curves for hollow cylinder metasurface designs in the case of using (**a**) Au, (**b**) Ag, (**c**) Al, and (**d**) TiN nanoantennas.

On the other hand, Aluminum (Al) shows significant variations in absorption with height, displaying sharp and multiple resonances as shown in Fig. 4c. Al’s resonance sensitivity to geometry makes it an excellent candidate for UV plasmonics, optical filtering, and dynamic metasurface applications. Compared to noble metals, aluminum remains a cost-effective alternative, but its overall absorption performance is weaker than TiN. In comparison with the hollow square metasurface, the hollow cylinder structure enhances localized plasmonic interactions, leading to more resonance peaks and broader absorption control. The pronounced redshifts in Au and Ag suggest that cylindrical geometries introduce additional Fabry-Pérot-like resonance effects^36^, which are less dominant in the hollow square structure. The cylindrical geometry supports longitudinal plasmonic modes that resemble Fabry–Pérot-like resonances, where the resonance condition depends on the effective vertical cavity length. The height-dependent nature of noble metals in contrast to TiN’s stability reinforces TiN’s superiority for applications requiring broadband, fabrication-tolerant absorbers. In Fig. 5, we present the average absorption curves for the hollow cylinder antenna metasurface using Au, Ag, Al, and TiN across different antenna heights (60 to 140 nm) reveal the impact of height variations on broadband absorption performance. As cleared from Fig. 5d, Despite changes in height, TiN consistently showed high absorptance, underscoring its robustness for cylindrical designs. In Fig. 5a, Gold (Au) exhibits a nearly constant absorption profile with a slight increase in absorption at intermediate heights (~ 80–100 nm), stabilizing around 0.7. This behavior suggests that Au-based metasurfaces allow for some tunability while retaining strong absorption, making them suitable for plasmonic sensors and optical filters.

Silver (Ag) in Fig. 5b shows a decreasing absorption trend with increasing height, indicating that Ag-based hollow cylinder metasurfaces experience a redshift in resonance, leading to reduced broadband absorption. This sensitivity to height suggests that Ag is more effective in designs requiring narrowband absorption or tunable spectral response. Finally in Fig. 5c, Aluminum (Al) also exhibits a gradual decline in absorption, stabilizing at lower values (~ 0.45), which aligns with its weaker plasmonic response in the visible range compared to Au and Ag. However, Al remains a cost-effective alternative for UV plasmonics and color filtering applications. When compared to the hollow square metasurface, the hollow cylinder design results in slightly more stable average absorption for Au and TiN, while Ag and Al show more sensitivity to height variations. These findings confirm that TiN is the best choice for broadband, height-independent absorption, while noble metals like Au and Ag provide more tunability for resonance-based applications.

### Cone metasurface

**Fig. 6:**
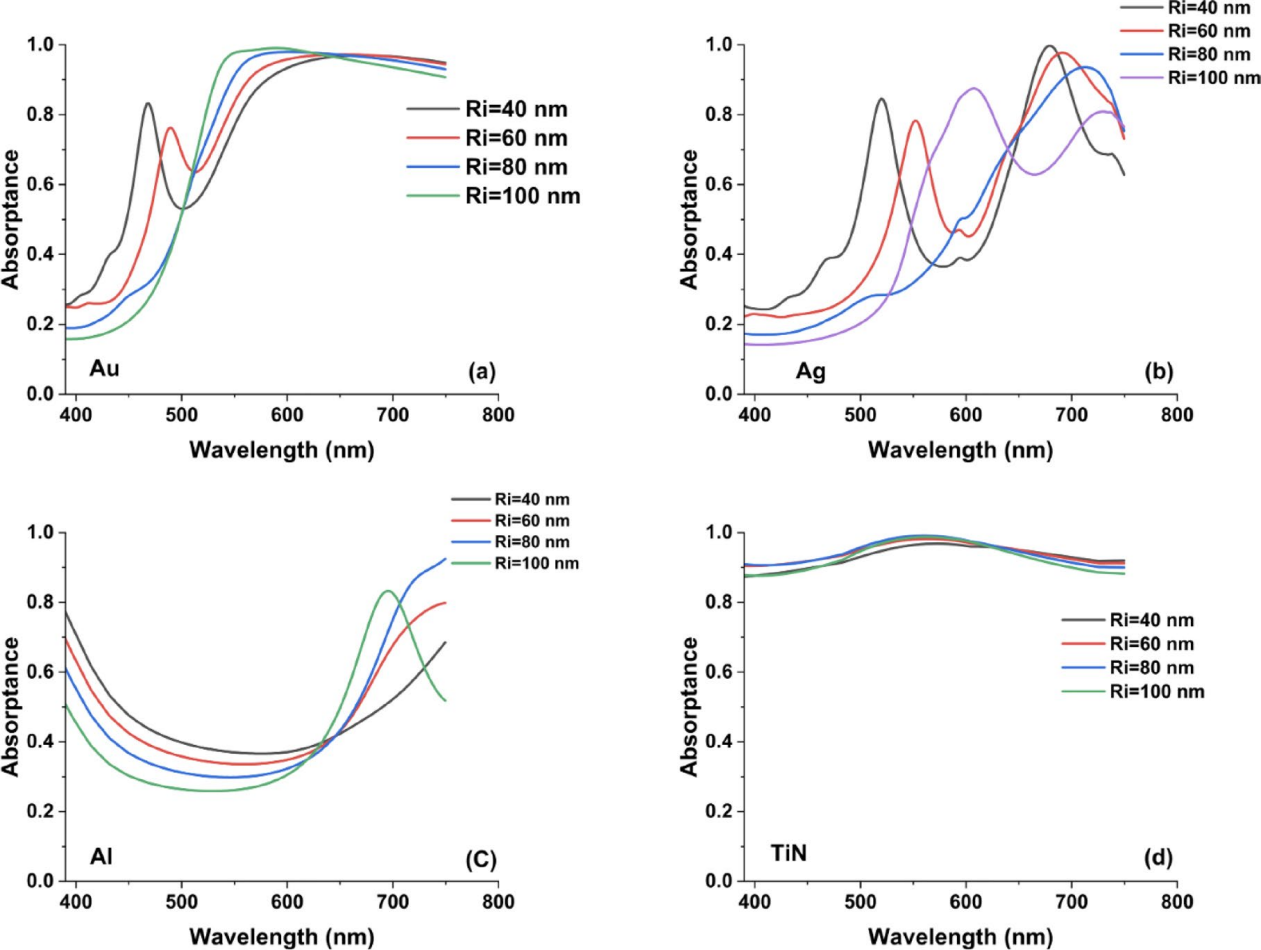
The resulted absorptance spectra in the case of cone metasurface designs by changing the upper radii by 20 nm step from Ri = 40 to 100 nm while keeping the bottom radius and height constant.

Figure 6 shows the absorption spectra for the cone antenna metasurface with varying upper radii (Ri = 40 nm, 60 nm, 80 nm, 100 nm) while keeping the bottom radius and height constant reveal key insights into the impact of upper radius tuning on plasmonic absorption. The results indicate significant variations in absorption intensity and spectral positioning across different materials (Au, Ag, Al, and TiN).

For Titanium Nitride (TiN) in Fig. 6d, the absorption remains almost constant and close to unity across the entire visible spectrum, suggesting that TiN’s broadband absorption is largely independent of upper radius variations. This behavior aligns with previous studies where TiN exhibits high optical loss and a strong plasmonic response, making it ideal for energy-harvesting and thermal applications. In contrast, Au and Ag exhibit multiple resonance peaks that shift with changing Ri, indicating strong localized surface plasmon resonance (LSPR) dependence on upper radius. Au reaches peak absorption near 500–600 nm, while Ag demonstrates strong absorption at longer wavelengths (~ 600–750 nm), making them suitable for wavelength-selective applications such as color filtering and biosensing.

In Fig. 6c, aluminum (Al) exhibits a relatively broad absorption band featuring a shallow minimum near 500 nm, which undergoes a slight spectral shift as the upper radius (Ri) increases. This behavior indicates that Al is particularly responsive to geometric modifications, making it suitable for tunable plasmonic configurations in the UV–visible range.

When compared with hollow square and hollow cylindrical metasurfaces, the conical nanoantenna geometry provides enhanced spectral adjustability. This effect is especially pronounced for noble metals (Au, Ag, and Al), where variations in the upper radius induce more substantial LSPR peak shifts due to the tapered profile and modified charge distribution along the antenna body.

**Fig. 7:**
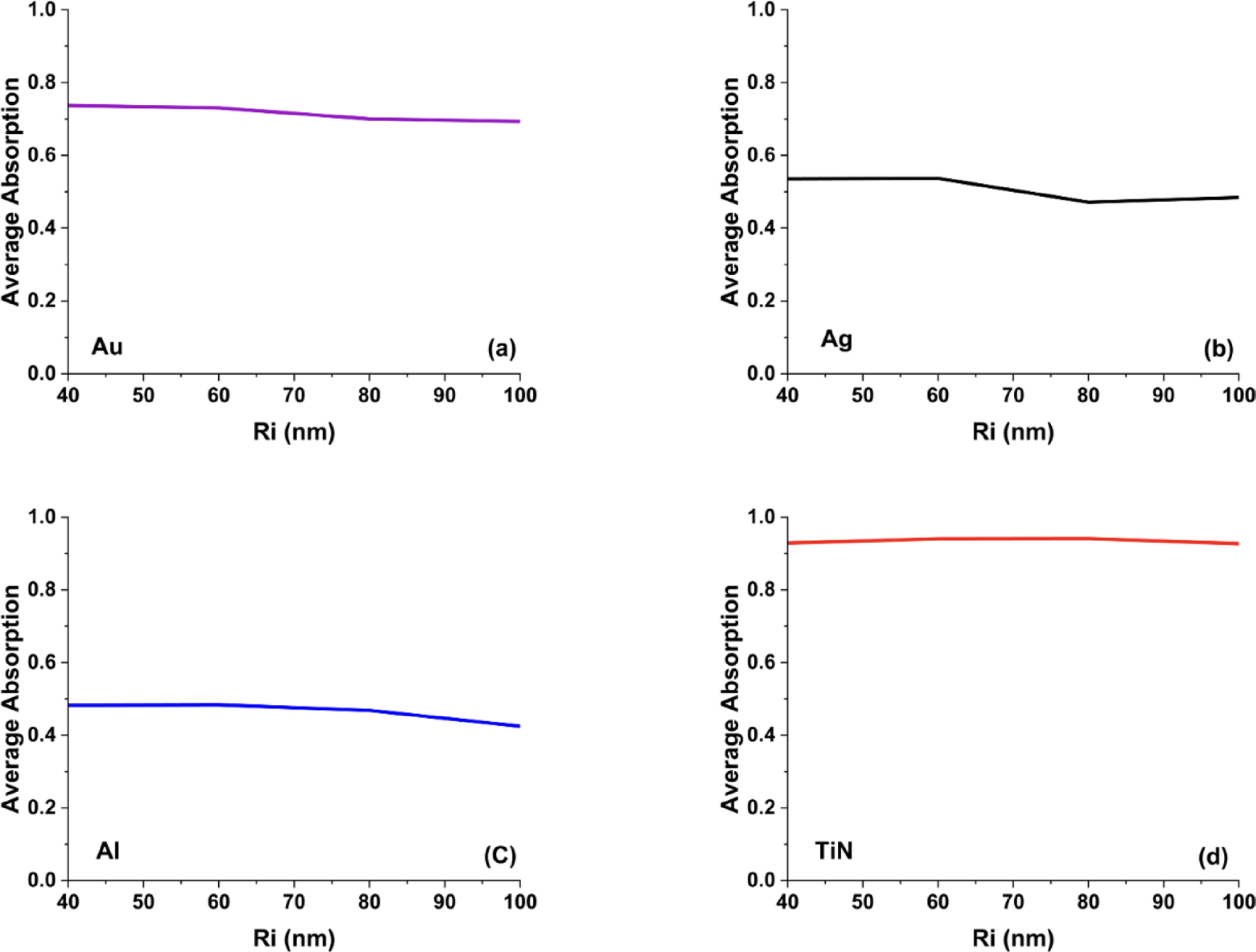
The calculated average absorption curves for cone metasurface designs by changing the upper radii in the case of using (**a**) Au, (**b**) Ag, (**c**) Al, and (**d**) TiN nanoantennas.

The average absorption spectra of the conical metasurface for Ri values ranging from 40 to 100 nm (Fig. 7) further illustrate the influence of top-radius variation on broadband absorptance across Au, Ag, Al, and TiN. In contrast to noble metals, Fig. 7d demonstrates that TiN maintains nearly unchanged absorption characteristics despite upper-radius modification, confirming its weak sensitivity to top-edge geometry and reinforcing its robustness against fabrication tolerances within the studied parameter space.

In contrast, Au and Ag in Fig. 7a, b exhibit a progressive reduction in average absorption as the upper radius (Ri) increases, indicating that enlarging the top radius induces a redshift accompanied by diminished absorption intensity across certain wavelength regions. This attenuation is more significant for Ag than for Au, reflecting Ag’s greater sensitivity to geometrical modifications. For Al, as shown in Fig. 7c, a moderate decrease in average absorption is observed with increasing Ri, suggesting that aluminum-based metasurfaces experience a gradual weakening of their overall plasmonic response as the upper radius expands. This behavior further implies that Al may be better suited for ultraviolet applications, where precise and sharper resonance tuning is advantageous.

Compared with hollow square and hollow cylindrical configurations, the conical nanoantenna design provides superior tunability of absorption characteristics—particularly for noble metals—since variations in the upper radius exert a direct and pronounced influence on resonance dynamics.

**Fig. 8:**
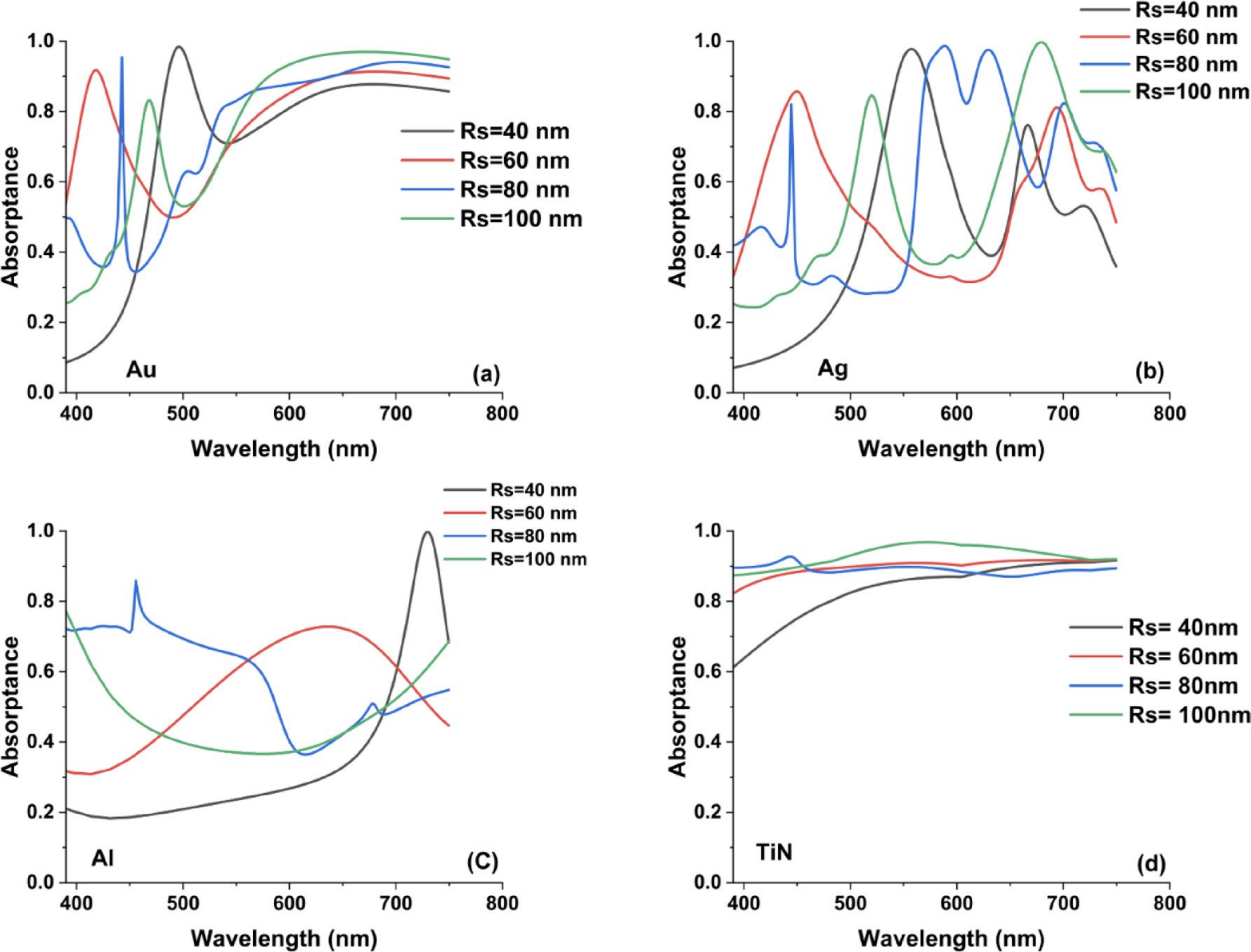
The resulted absorptance spectra in the case of cone metasurface designs in the case of using (**a**) Au, (**b**) Ag, (**c**) Al, and (**d**) TiN nanoantennas by changing the bottom radii by 20 nm step from Rs = 40 to 100 nm while keeping the upper radius and height constant.

Figure 8 presents the absorption spectra of the conical nanoantenna metasurface for different bottom radii (Rs = 40, 60, 80, and 100 nm), while keeping the top radius and height fixed. This analysis highlights how variations in the base dimension influence plasmonic absorption characteristics. The results demonstrate that the bottom radius plays a crucial role in shaping the resonance response and spectral absorption behavior for different materials (Au, Ag, Al, and TiN).

For Titanium Nitride (TiN), as illustrated in Fig. 8d, the absorptance remains nearly unity and exhibits minimal spectral variation across the visible range. This behavior indicates that TiN-based metasurfaces provide broadband and structurally tolerant absorption with weak sensitivity to base-radius changes. Such performance aligns with previous studies reporting that TiN’s relatively high intrinsic losses and broad plasmonic damping enable stable absorption, making it a practical alternative to noble metals in applications such as thermal emitters and energy harvesting systems^37^.

In contrast, gold (Au) and silver (Ag) display pronounced changes in absorption as Rs increases, reflecting a strong dependence on localized surface plasmon resonance (LSPR) effects. In particular, Au (Fig. 8a) exhibits well-defined resonance peaks within the 450–650 nm range, where both peak position and intensity evolve with increasing bottom radius. This confirms the high tunability of noble-metal-based metasurfaces and their effectiveness for wavelength-selective plasmonic applications.

From Fig. 8b, we can notice that Silver (Ag) displays multiple resonant peaks that shift and broaden with increasing Rs, suggesting a complex interaction between LSPR and propagating surface plasmon modes. The strong tunability of Ag metasurfaces makes them attractive for biosensing and optical filtering applications. Aluminum (Al) in Fig. 8c exhibits significant variations in absorptance, with peaks shifting toward longer wavelengths as Rs increases. The broad and tunable absorption response of Al-based cone metasurfaces suggests potential applications in UV plasmonics and photodetection. Compared to hollow square and hollow cylinder metasurfaces, the cone antenna design provides enhanced spectral tunability, particularly for noble metals, where the bottom radius significantly alters plasmonic resonance characteristics.

**Fig. 9:**
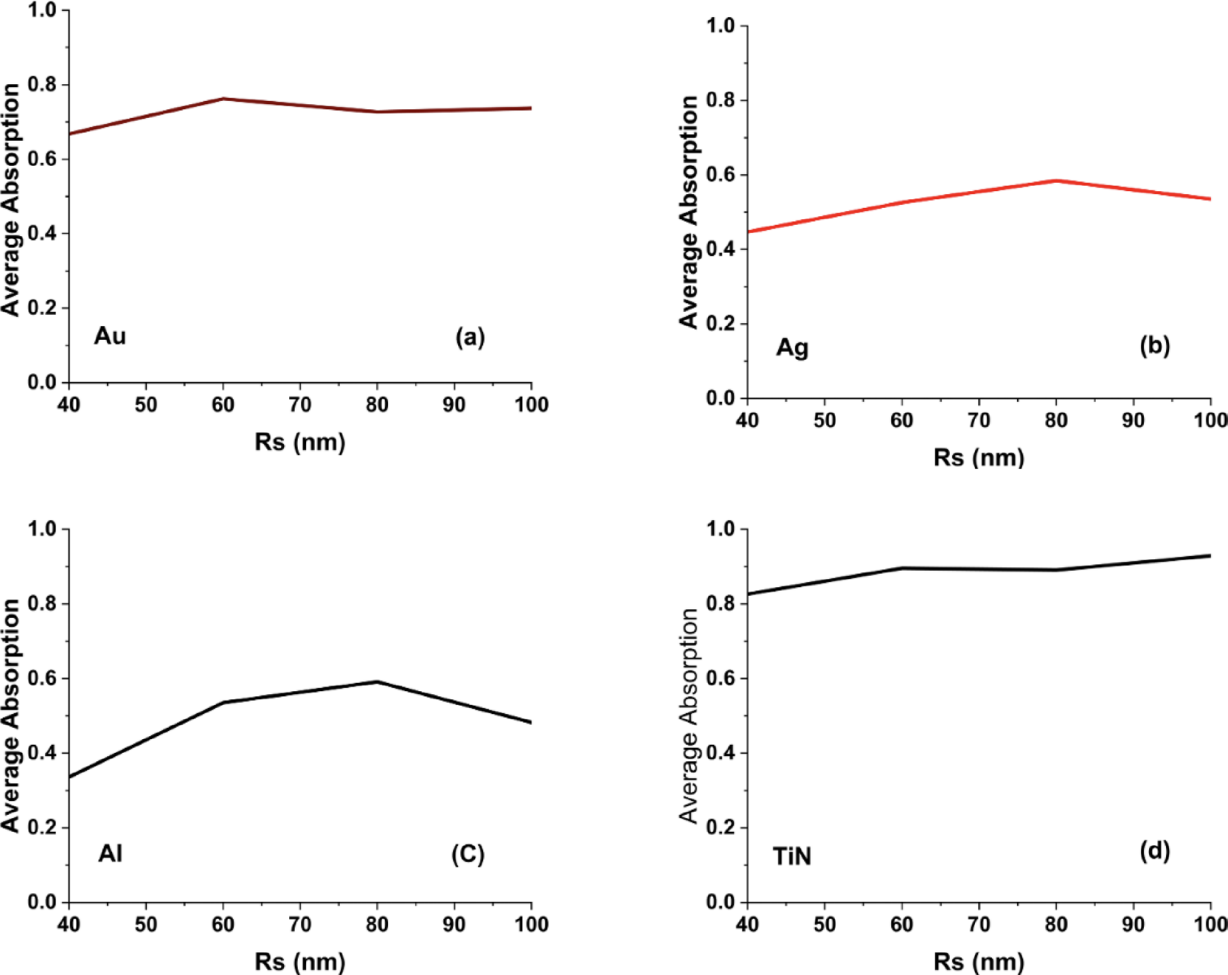
The calculated average absorption curves for cone metasurface designs by changing the bottom radii in the case of using (**a**) Au, (**b**) Ag, (**c**) Al, and (**d**) TiN nanoantennas.

The average absorption curves for Au, Ag, Al, and TiN cone antennas with varying bottom radii (Rs = 40 to 100 nm) in Fig. 9 highlight how geometric tuning impacts broadband absorption. In Fig. 9a, Bottom radius tuning had minimal effect on TiN, making it suitable for broadband absorption with base-level design flexibility. In addition, Fig. 9a shows that Gold (Au) exhibits a gentle increase in average absorption with Rs, stabilizing at around 0.70, indicating that modest geometric adjustments can fine-tune Au’s plasmonic behavior without drastically altering its broadband response.

On the other hand, Silver (Ag) and Aluminum (Al) in Fig. 9b, c, respectively show more pronounced peaks and dips in their average absorption as Rs varies, suggesting higher sensitivity to localized surface plasmon resonance (LSPR) shifts. Ag peaks around intermediate bottom radii, then slightly declines, a sign that Ag-based cone metasurfaces can be optimized for narrowband absorbers or sensors. Meanwhile, Al peaks in the mid-range of Rs but drops off at the largest radius, highlighting its weaker visible-range plasmonic performance yet potential for UV plasmonics and cost-sensitive applications . Overall, these trends confirm that TiN provides the most robust broadband absorption, while Au, Ag, and Al offer varying degrees of spectral tunability depending on the target application.

## Fitting models and predictive analysis

**Fig. 10:**
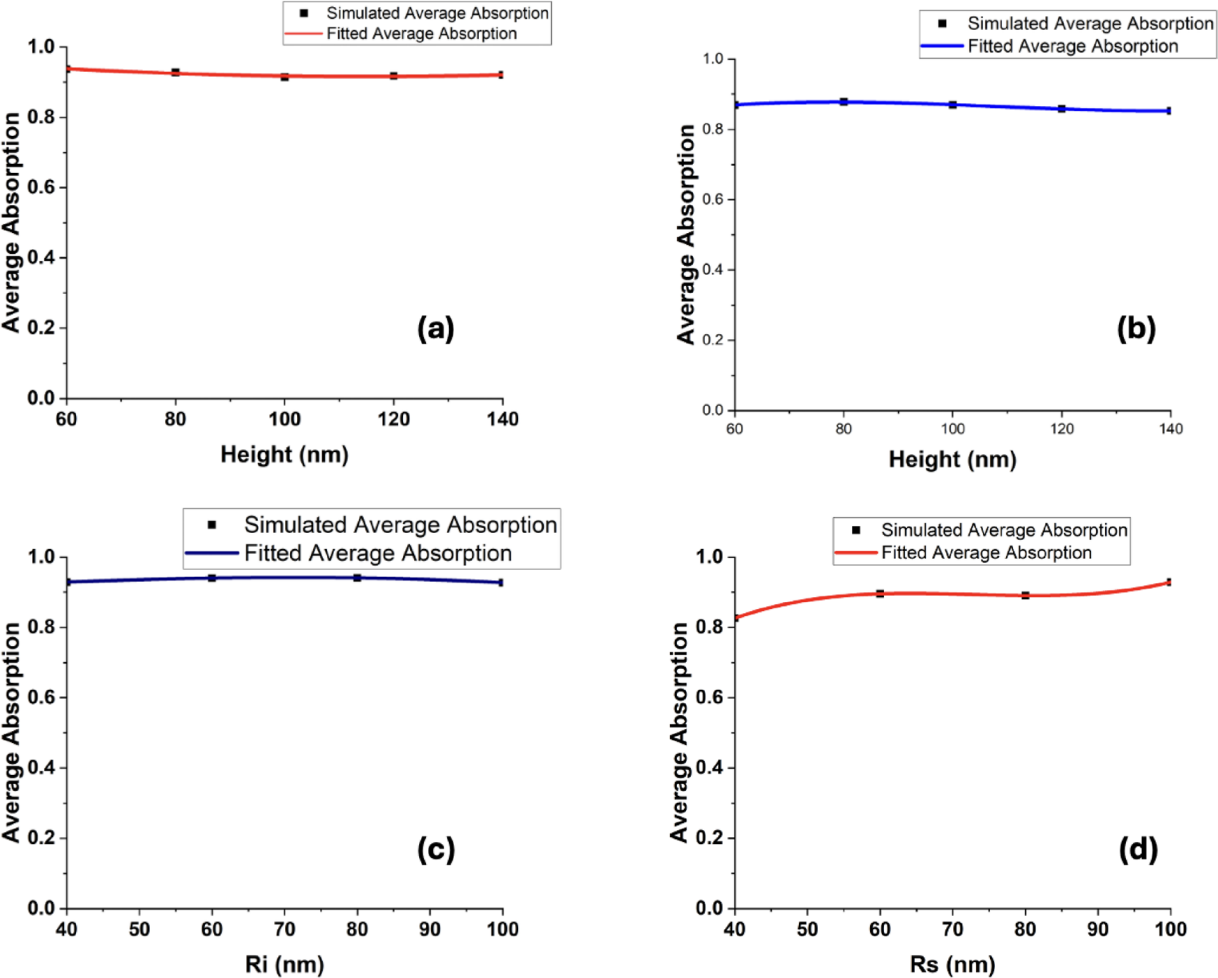
The change of the average absorption with (**a**) the hollow square height for the TiN metasurface design, (**b**) the hollow cylinder height, (**c**) with the hollow cylinder upper radius and (**d**) with the hollow cylinder bottom radius for the TiN metasurface design. The dots represent simulated data while solid lines show the fitted data.

Finally, regression analysis was performed for the TiN-based metasurface configurations incorporating hollow square and hollow cylindrical nanoantennas. A third-order polynomial model was adopted, as it represents the simplest fitting function capable of accurately capturing the simulated average-absorption behavior across the studied geometrical intervals. It should be emphasized that these empirical expressions are restricted to the defined design parameter ranges and are not intended for extrapolation beyond those limits. Figure 10 illustrates the corresponding fitted relationships describing the variation of the average absorptance with: (a) the hollow square antenna height in the TiN metasurface configuration, (b) the hollow cylinder height, (c) the upper radius of the hollow cylinder, and (d) the lower radius of the hollow cylinder within the TiN design framework. The simulated results show a nearly flat absorption response around 0.94 across varying antenna heights, suggesting excellent design robustness. This minimal variation is due to the strong inherent plasmonic response of TiN and its broadband loss characteristics. As the antenna height increases from 60 to 140 nm, the effective optical path length changes only slightly, leading to negligible alteration in the field confinement. The flatness of the curve also implies that this geometry is less sensitive to fabrication errors. The fitting curve closely matches the simulated points, supporting the validity of a quadratic or low-order polynomial model for height-dependence. Such behavior is ideal for practical deployment in thermal management systems, where dimensional consistency may be difficult to maintain. This makes the hollow square TiN metasurface a highly attractive design for robust absorber platforms.

Figure 10b illustrates the variation of average absorptance as a function of antenna height for the hollow cylindrical TiN metasurface. The average absorption initially approaches 0.88 and then exhibits a slight reduction as the height increases up to 140 nm. This gradual decrease can be attributed to enhanced radiative damping in taller structures, which modifies the characteristics of the localized surface plasmon resonance. Nevertheless, the overall change remains modest, demonstrating that the absorption response is relatively robust against height variations. Compared to noble metals, TiN maintains a more stable absorption profile across a wider geometric range, largely due to its substantial imaginary permittivity component. The curve fitting analysis reveals a nonlinear dependence, which can be approximated by a weak parabolic trend. Such predictable behavior is advantageous for structural optimization, allowing designers to fine-tune antenna height according to application requirements. Moreover, the slight absorption reduction can be mitigated through adjustments to the dielectric spacer thickness or the properties of the metallic back reflector, since the plasmonic response is governed by the global cavity resonance. Overall, the hollow cylindrical configuration achieves a balanced trade-off between geometrical tunability and broadband absorption efficiency.

Then, the dependence of the average absorptance on the upper radius (Ri) of the hollow cylindrical TiN metasurface is illustrated in Fig. 10c. It is observed that the mean absorption remains nearly constant—slightly exceeding 0.92—as Ri increases from 40 to 100 nm. This behavior suggests that variations in the upper radius exert only a negligible influence on the plasmonic resonance characteristics or coupling efficiency of the TiN antenna. Moreover, the almost flat fitting curve confirms that electromagnetic energy is predominantly confined near the base or central region of the structure, rendering modifications at the upper rim comparatively insignificant in shaping the overall absorption performance.

Such insensitivity is a valuable trait for sensors or devices operating under mechanical or thermal stress. This behavior can be theoretically linked to the minor participation of the upper rim in mode localization due to the asymmetric geometry. The fitted trend shows excellent agreement with simulation points, affirming that geometric tuning via Ri is not the dominant mechanism for tuning absorption. These findings promote the use of top-insensitive geometries in scalable absorber fabrication processes.

Finally, the dependence of the fitted average absorptance on the bottom radius (Rs) of the hollow cylindrical TiN metasurface was examined, as illustrated in Fig. 10d. Increasing Rs from 40 to 100 nm leads to a noticeable rise in the mean absorption, from approximately 0.82 to about 0.92. This trend indicates that the base radius plays a crucial role in governing electromagnetic confinement and dissipative losses within the structure. Larger Rs values strengthen the coupling between the incident wave and the resonator, thereby promoting more efficient plasmonic excitation and enhanced energy absorption in the TiN nanoantenna.

The observed behavior can be attributed to the enlarged effective interaction area and the strengthened dielectric–metal coupling, where broader base dimensions facilitate the excitation of higher-order resonant modes. The fitted response curve exhibits an upward convex profile, indicating that the base radius (Rs) plays a significant role in tailoring broadband absorption characteristics. Such a configuration is particularly advantageous for applications demanding stable high absorptance over wide spectral ranges, including solar energy harvesting and thermophotovoltaic systems. Moreover, it provides a flexible platform for impedance matching between the absorber and its surrounding medium. These findings highlight the importance of geometrical base optimization in achieving high-efficiency metasurface absorbers. Although the current investigation is based exclusively on finite element method (FEM) simulations, the results demonstrate strong consistency—and in certain aspects measurable enhancement—relative to comparable studies reported in the literature, as summarized in Table 1.

**Table 1:** Comparison with previous works.

References	Geometry/design	Simulation method	Spectral range (nm)	Average absorption (%)
Zeng et al.^38^	Multilayer refractory TiN metasurface	FDTD	300–2500	91.5
He et al.^39^	Omnidirectional TiN metasurface absorber	Numerical (FDTD/FEM)	Visible–NIR	~ 85
Smith et al.^40^	Ultrathin TiN metasurface absorber	Numerical	Visible	~ 86
Liu et al.^41^	Cross-shaped TiN metasurface	Numerical	Visible–NIR	~ 90
This work	Hollow square/cylinder/cone TiN metasurfaces	Numerical (FEM)	400–800	**~ 90–93**

Regarding the fitting part, we found that the average absorption ${A}_{sqaure}$ and ${A}_{hollow cylinder}$ for square and hollow cylinder antennas respectively, depend on the geometry of the utilized nanoantennas can be fitted and represented by the following equations for hollow square height (${h}_{1}$), hollow cylinder height (${h}_{2}$), hollow cylinder upper radius (${r}_{i}$), and hollow cylinder bottom radius (${r}_{s}$), respectively as follow;

1 $$A_{sqaure} = a_{1} h_{1}^{3} + b_{1} h_{1}^{2} + c_{1} h_{1} + d_{1}$$

2 $$A_{hollow cylinder} = a_{2} h_{2}^{3} + b_{2} h_{2}^{2} + c_{2} h_{2} + d_{2}$$

3 $$A_{hollow cylinder} = a_{3} r_{i}^{3} + b_{3} r_{i}^{2} + c_{3} r_{i} + d_{3}$$

4 $$A_{hollow cylinder} = a_{4} r_{s}^{3} + b_{4} r_{s}^{2} + c_{4} r_{s} + d_{4}$$

We have used the cubic fit as it supports minimal coefficients for a given acceptable accuracy. The symbols a_1_, b_1_, c_1_, d_1_, a_2_, b_2_, c_2_, d_2_, a_3_, b_3_, c_3_, d_3_, a_4_, b_4_, c_4_ and d_4_ are the fitting coefficients and their values can be found in Table 2. The proposed metasurface structures, particularly those based on TiN, are compatible with current CMOS-compatible deposition techniques such as sputtering and atomic layer deposition (ALD). However, precise nanofabrication of hollow or conical antennas may require focused ion beam (FIB) or electron beam lithography (EBL), which presents challenges in scalability and cost. Future work may explore template-assisted methods or nanoimprint lithography as scalable alternatives. The average absorption was calculated from the simulated absorptance spectra obtained using COMSOL Multiphysics. For a given metasurface design, the absorptance $A\left( \lambda \right)$ was averaged over the considered wavelength range according to

$$\overline{A} = \frac{1}{{\lambda_{2} - \lambda_{1} }}\mathop \smallint \limits_{{\lambda_{1} }}^{{\lambda_{2} }} A\left( \lambda \right)d\lambda$$

where $\lambda_{1} = 400$ nm and $\lambda_{2} = 800$ nm define the visible spectral range investigated in this work. In practice, the absorptance spectra were exported from COMSOL and post-processed in MATLAB. The integral was evaluated numerically using the trapezoidal rule, corresponding to

$$\overline{A} \approx \frac{1}{{\lambda_{2} - \lambda_{1} }}\mathop \sum \limits_{i = 1}^{N - 1} \frac{{A\left( {\lambda_{i} } \right) + A\left( {\lambda_{i + 1} } \right)}}{2}\left( {\left( {\lambda_{i + 1} } \right) - A\left( {\lambda_{i} } \right)} \right)$$

ensuring consistent and reproducible calculation of the average absorption for all metasurface configurations.

**Table 2:** Fitting coefficients for different metasurface designs based on geometrical variations.

Metasurface design	Fitting coefficient	Value
Hollow square (change in height)	a_1_	− 0.0017
	b_1_	7.39304 × 10^−6^
	c_1_	− 3.89226 × 10^−21^
	d_1_	1.01355
Hollow cylinder (change in height)	a_2_	0.00764
	b_2_	− 7.65601 × 10^−5^
	c_2_	2.36081 × 10^−7^
	d_2_	0.63562
Hollow cylinder (change in upper radius)	a_3_	0.00115
	b_3_	6.325 × 10^−8^
	c_3_	− 7.61625 × 10^−8^
	d_3_	0.88772
Hollow cylinder (change in upper radius)	a_4_	0.03826
	b_4_	− 5.34123 × 10^−4^
	c_4_	2.45054 × 10^−6^
	d_4_	− 0.0064

## Conclusion

In this work, plasmonic metasurface absorbers based on hollow square, hollow cylindrical, and conical nanoantenna geometries were numerically investigated using different plasmonic materials, with particular focus on TiN. Within the studied geometries and visible spectral range, TiN-based designs exhibit relatively high average absorption, improved spectral stability, and lower sensitivity to geometric variations compared to noble metals. This behavior is mainly attributed to TiN’s intrinsic optical properties, including higher dissipative loss and broader plasmonic damping in the visible regime, which support broadband absorption with reduced dependence on sharp resonances. In contrast, Au and Ag show stronger resonance-dominated responses, while Al demonstrates higher sensitivity to geometric perturbations. Geometry-dependent fitted models were also developed to estimate average absorption from key structural parameters, identifying antenna height and base radius as the most influential factors. Although limited to numerical analysis, the results suggest TiN as a CMOS-compatible platform for broadband metasurface absorbers. Future work will include experimental validation and angular-response analysis.

## Data Availability

Requests for data and materials should be addressed to corresponding author e-mail: arafaaly@aucegypt.edu.
